# Hemoglobin-carbon nanotube derived noble-metal-free Fe_5_C_2_-based catalyst for highly efficient oxygen reduction reaction

**DOI:** 10.1038/srep20132

**Published:** 2016-02-03

**Authors:** Varun Vij, Jitendra N. Tiwari, Wang-Geun Lee, Taeseung Yoon, Kwang S. Kim

**Affiliations:** 1 Center for Superfunctional Materials, Department of Chemistry, Ulsan National Institute of Science and Technology (UNIST), UNIST-gil 50, Ulsan 689-798, Korea

## Abstract

High performance non-precious cathodic catalysts for oxygen reduction reaction (ORR) are vital for the development of energy materials and devices. Here, we report an noble metal free, Fe_5_C_2_ nanoparticles-studded sp^2^ carbon supported mesoporous material (**CNTHb-700**) as cathodic catalyst for ORR, which was prepared by pyrolizing the hybrid adduct of single walled carbon nanotubes (CNT) and lyophilized hemoglobin (Hb) at 700 °C. The catalyst shows onset potentials of 0.92 V in 0.1 M HClO_4_ and in 0.1 M KOH which are as good as commercial Pt/C catalyst, giving very high current density of 6.34 and 6.69 mA cm^−2^ at 0.55 V vs. reversible hydrogen electrode (RHE), respectively. This catalyst has been confirmed to follow 4-electron mechanism for ORR and shows high electrochemical stability in both acidic and basic media. Catalyst **CNTHb-700** possesses much higher tolerance towards methanol than the commercial Pt/C catalyst. Highly efficient catalytic properties of **CNTHb-700** could lead to fundamental understanding of utilization of biomolecules in ORR and materialization of proton exchange membrane fuel cells for clean energy production.

The essential role of ORR in proton exchange membrane fuel cells (PEMFC) and metal air batteries for enhanced industrial demand of high energy efficacy has increased its technological importance^1^,^2^,^3^. Despite being exorbitant and less abundant, noble metals like platinum and gold and its alloys have been long relied on for development of cathodic catalysts for ORR on account of their low overpotential and fast reduction kinetics^4^,^5^,^6^,^7^. However, due to high cost of these noble metals, there have been many attempts to develop non-precious metal-nitrogen/carbon (M-N/C) composites in which these pricey metals are replaced with cheap but electrochemically active transition metals like Fe and Co and their alloys^8^,^9^,^10^,^11^,^12^,^13^,^14^. Nitrogen, on the other hand, enhances the polarization of porous surface due to its basic nature, which results in high degree of surface adsorption of oxygen and thus assists ORR. As such, the development of commercially viable catalysts with high ORR properties, derived from inexpensive and naturally abundant precursors, has been a great challenge in the field of clean energy production. In this regard, use of selective transition metal carbide nanoparticles as catalytic sites on conductive carbon materials with high surface area can be envisioned as promising materials due to high surface activity and high durability.

Recently, the development of nanomaterials using metalloproteins with good electron transfer capabilities has attracted particular interest^15^,^16^,^17^. Among various transition metal compounds, metal carbides have been widely studied for their application as electrochemical catalysts due to their high conductivity and high resistance for corrosion^18^,^19^. The accumulation of appropriate portion of carbon can effectively modify the electron distribution in a metal to change its electronegativity in favor of its catalytic properties^20^. Herein, we have synthesized an sp^2^ carbon supported mesoporous material **CNTHb-700** uniformly decorated with iron carbide (Fe_5_C_2_) nanoparticles by pyrolyzing the hybrid adduct of most common metalloprotein Hb and oxidized CNTs in the presence of ammonia gas. Hb with a quaternary structure, plays a dual role in synthesis of carbonized M-N/C material; (i) inherent Fe^2+^ ions located in each of four heme groups of a Hb molecule serve as a source of ORR active Fe_5_C_2_ nanoparticles-based catalytic sites and (ii) polypeptide chains and pyrrolic units of heme group behave as precursors for nitrogen doped sp^2^ carbon support. We observed that these Fe_5_C_2_ (510) nanoparticles behave as efficient oxygen reduction sites, showing the onset potential as good as commercial catalyst (20% Pt/C), while highly conducting Fe_5_C_2_ nanoparticles lead to efficient charge transfer responsible for high current density. We discuss the outstanding electrochemical features of the cost effective catalyst showing remarkable stability in its current density even after 10000 voltammetric cycles.

Earlier, blood powder has been used to generate nitrogen doped carbonized supporting material for oxygen reduction properties in only basic medium^21^. Due to relatively very low percentage of Fe (heme groups) in blood than in pure hemoglobin, main catalytic sites are either ‘doped heteroatoms’ generated by carbonized proteins of blood plasma like albumins, globulins and fibrinogens or/and extrinsically incorporated metal based nanoparticles^21^. In **CNTHb-700**, Hb molecules are self sufficient to generate Fe_5_C_2_ catalytic sites without any need of extrinsic addition of metal based nanoparticles for ORR properties in both acid and base. To best of our knowledge, this is the first report on synthesis of Fe_5_C_2_ nanoparticles appended CNTs from Hb/CNT adduct for the development of cathodic electrode for excellent oxygen reduction reaction in both acidic as well as in basic media.

## Results

**Figure 1:**
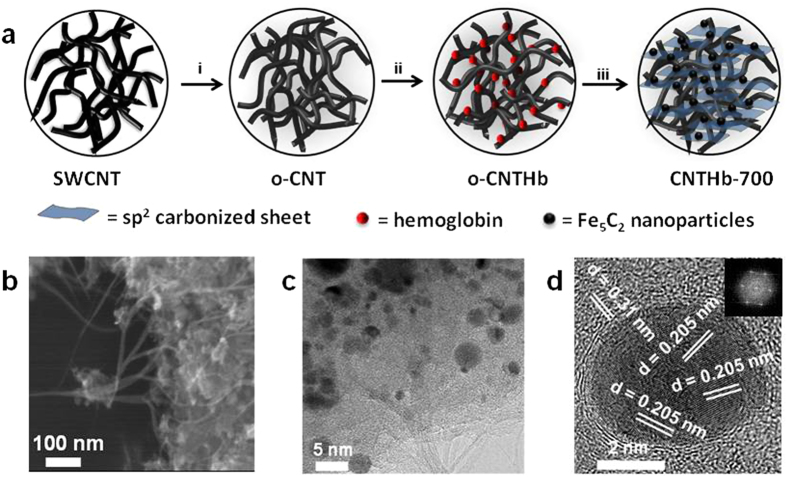
Synthesis procedure and morphology characterization for CNTHb-700. (**a)** Schematic diagram of the synthetic procedure: (i) H_2_SO_4_:HNO_3_ (7:3), (ii) aqueous solution of hemoglobin (Hb). (iii) pyrolysis under NH_3_/N_2_ followed by leaching with 0.1 M H_2_SO_4_. (**b**) SEM and (**c**,**d**) HRTEM images (inset: FFT pattern of Fe_5_C_2_ nanoparticles).

The single walled CNT (SWCNT) was oxidized to oxidized CNT (o-CNT) in the presence of piranha according to a previous report^22^. To the well dispersed o-CNT in distilled water (60 mg/50ml), an aqueous solution of 40 mg of lyophilized Hb was added dropwise at 4 °C to avoid early denaturation of protein. The dispersion was allowed to be stirred for overnight at room temperature. The dispersion was centrifuged and washed with water 4–5 times. The solid part was dried and then placed in quartz pyrolysis tube and heated at 700 °C for 2 h with a rate of temperature increase 9 °C/min in NH_3_ atmosphere to give nitrogen doped carbonized composite **CNTHb-700**. The product was leached for 3 h in 0.1M sulfuric acid to remove inactive metal particles. Other catalysts (**CNTHb1**, **CNTHb-II** and **CNTHb-III**) were synthesized using same procedure by varying the amount of Hb in o-CNT. A schematic diagram shows the stepwise procedure for the synthesis of composite **CNTHb-700** (Fig. 1a).

The scanning electron microscopy (SEM) of **CNTHb-700** shows the formation of Fe_5_C_2_ nanoparticles over CNTs (Fig. 1b). High resolution (HR) transmission electron microscopic (TEM) (HRTEM) images of **CNTHb-700** confirm the uniform adsorption of Fe_5_C_2_ nanoparticles along with carbonized sheets over CNTs (Fig. 1c). The distribution of Fe_5_C_2_ nanoparticles was supported by TEM-mapping of electronic image of catalyst **CNTHb-700** (Supporting Information Fig. S1). The nanoparticles in HRTEM image possess lattice spacing of 0.205 nm (Fig. 1d) which clearly corresponds to Fe_5_C_2_ (510)^23^, whereas the carbonized part of Hb with lattice spacing of 0.31 nm corresponds to lattice fringes of C (002) planes, confirming the sp^2^ character^24^. The crystallanity of Fe_5_C_2_ nanoparticles was confirmed by fast Fourier transform (FFT) pattern (Inset, Fig. 1d) which exhibits the C_3_ symmetric structure of nanoparticles. The nanoparticles size-distribution study of a wider TEM image (Supporting Information Fig. S2a) with ~130 nanoparticles showed that ~63% of these particles lie below the range of 5 nm of diameter at the surface, whereas approximately 35% particles have diameter in the range of 2.0–4.0 nm (Supporting Information Fig. S2b). Such ultrafine nanoparticles are the reason of good dispersion of nanoparticles and more uniform adsorption over the carbon material.

Being a metalloprotein, Hb irreversibly adsorbs at the oxidized surface of oxidized CNTs (o-CNTs) due to hydrophobic interactions which further results in direct electron transfer (DET) interactions between protein and carbon materials to give oxidized CNTHb (o-CNTHb) hybrid adduct^25^. The UV-vis spectral study (Supporting Information Fig. S3) shows the interactions between oxidized SWCNT and Hb.

The o-CNT shows an undefined absorption band at 200–250 nm range, whereas the Hb in its aqueous solution shows the characteristic Soret band at 405 nm. Upon addition of aqueous solution of aqueous hemoglobin to aqueous dispersion of o-CNT, a defined band at 209 nm was observed along with another band at 405 nm which indicates the interactions between o-CNT with Hb in o-CNTHb adduct (Supporting Information Fig. S3). However, no change in characteristic Soret band corresponding to Hb at 405 nm was observed which means that secondary structure of protein is unperturbed^26^. Besides, the addition of Hb to CNT leads to immediate precipitation of otherwise soluble o-CNT and Hb solutions due to alteration in surface morphology of material by strong interactions between them (Supporting Information Fig. S3, Inset).

The electron transfer between Hb and o-CNT was further confirmed by comparing Raman spectra of o-CNT and o-CNTHb (Supporting Information Fig. S4). The G band corresponding to o-CNT is observed at 1596 cm^−1^ which shifts to the lower value of 1580 cm^−1^ upon addition of Hb, confirming the electron transfer. In addition to this, the 2D or G’ band in o-CNTHb is observed at higher wavenumber as compared to that in o-CNT, which indicates the separation of CNTs due to the increased negatively charged electron density on CNTs upon adsorption of Hb molecules on their surfaces. The decreased intensity of D band in o-CNTHb is possibly due to the denaturation of Hb molecules adsorbed on o-CNT. However, the Raman spectrum of **CNTHb-700** shows enhancement in intensity of D band, thus leading to high I_D_/I_G_ ratio which indicates the increased numbers of surface defect attributed to adsorbed iron carbide nanoparticles and nitrogen doping on the CNT surfaces^27^.

In powder X-ray differaction (XRD) pattern of **CNTHb-700**, the prominent peak at 26.69° can be attributed to the sp^2^ C(002) of CNT. Other small peaks at 2θ value 44.12° 51.45° and 75.66° correspond to sp^2^ carbons C(100), C(004) and C(110). Same diffraction peaks can also be observed in the XRD spectrum of oxidized o-CNT. In comparison to o-CNT, much stronger diffraction peak can be observed at 44.17° in the case of **CNTHb-700** due to Fe_5_C_2_ nanoparticles with the lattice planes Fe_5_C_2_ (510) (Supporting Information Fig. S5)^23^.

FT-IR spectrum of **CNTHb-700** shows broad stretching bands at 1137 cm^−1^ and 1564 cm^−1^ are characteristic features of C-N bond and C=O/C=N bonds, which indicates the high extent of nitrogen doping in material (Supporting Information Fig. S6). No significant band corresponding to C-N and C=N was observed in IR spectrum of o-CNT. The band at 2914 cm^−1^ corresponds to C-H bond stretches.

**Figure 2:**
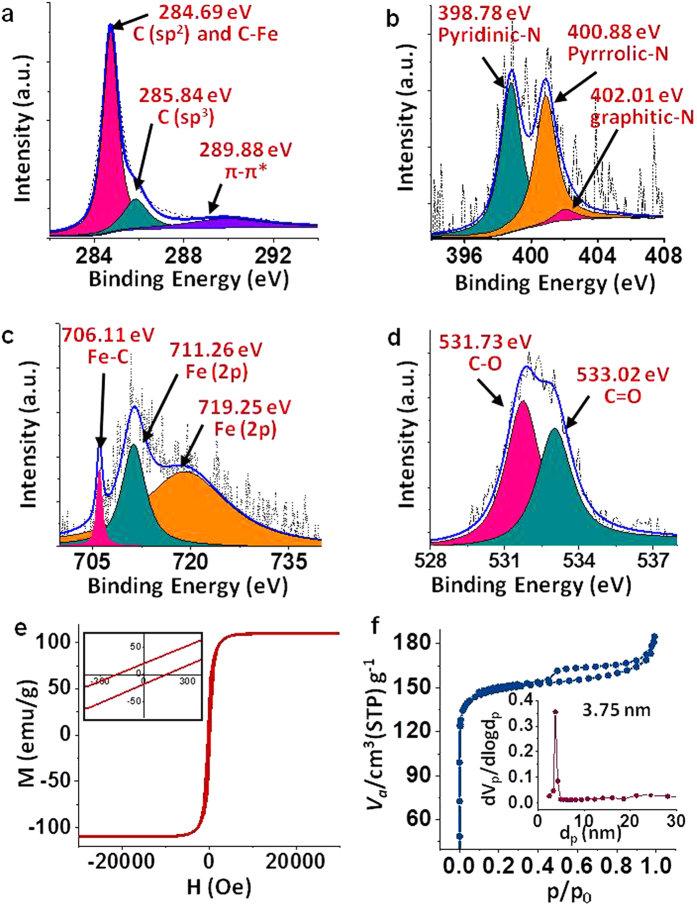
XPS, Magnetometeric analysis and BET isotherm. (**a**) C 1s, (**b**) N 1s, (**c**) Fe 2p, (**d**) O 1s of **CNTHb-700** catalyst. (**e**) Magnetic hysteresis curves corresponding to **CNTHb-700** (0.7 mg) at 300 K. The magnetization value is with respect to per gram of Fe_5_C_2_ nanoparticles. (**f**) BET isotherm of the **CNTHb-700** catalyst showing the mesoporous state; Inset shows Barrett-Joyner-Halenda (BJH) algorithm spectrum for the calculation of average pore diameter of the mesoporous **CNTHb-700** catalyst.

The key elements (C, N, O and Fe) in **CNTHb-700** were determined by wide scan XPS spectra (Supporting Information Fig. S7). The C1s peak is unraveled into three peaks at 284.69 eV, 285.84 eV and 289.88 eV. The peak at 284.69 eV is a merged signal corresponding to sp^2^ carbon and C-Fe bond, whereas peaks at 285.84 eV and 289.88 eV are attributed to sp^3^ carbon and π − π* interactions, respectively (Fig. 2a)^28^. The N1s peak shows pyridinic, pyrrolic and graphitic nitrogen at 398.78 eV, 400.88 eV and 402.01 eV, respectively (Fig. 2b)^29^. In the case of Fe (2p) (Fig. 2c), the peaks at 706.11 eV and 719.25 eV confirms the formation of Fe_5_C_2_ nanoparticles, whereas the peak at 711.26 eV can be assigned to the binding energy of 2p_3/2_ orbital of Fe^3+^ moieties^23^,^30^. This result corroborates with the XRD results. For O1s, two peaks at 531.73 eV and 533.02 eV were observed due to C-O and C=O, respectively (Fig. 2d)^31^. However, the composition of the material was confirmed by elemental analysis (for C, N and O) and ICP-OES analysis (for Fe) which showed the weight % of C, N, O and Fe to be 78.48%, 7.25%, 8.2% and 5.8%, respectively.

The magnetic hysteresis loop of **CNTHb-700** was recorded at 300 K to confirm the formation of Fe_5_C_2_ nanoparticles. The magnetization value was calculated with respect to per gram of Fe_5_C_2_ nanoparticles. It shows saturation magnetization value of 105 emu g^−1^ (Fig. 2e) which is very close to that of the characteristic value of soft ferro/ferrimagnetic Fe_5_C_2_ nanoparticles^23^. The magnetic intensities are little lower than bulk Fe_5_C_2_ due to the presence of CNTs and carbonized sp^2^ material.

The Brunauer-Emmett-Teller (BET) study of adsorption-desorption isotherm resulted in the curve supporting mesoporous phase in **CNTHb-700** (Fig. 2f) with surface area of 459.25 m^2^g^−1^ and average volume of 105.51 cm^3^g^−1^. The pore size distribution in **CNTHb-700** was studied by the Barrett-Joyner-Halenda (BJH) algorithm for approximation using nitrogen desorption branch at 77 K which revealed that most of the pores have diameter of 3.75 nm making the material highly mesoporous (Fig. 2f, Inset). The mesoporous material with such high surface area provides a plenty of catalytic surface for ORR, which also justifies the high catalytic performance of **CNTHb-700** catalyst.

**Figure 3:**
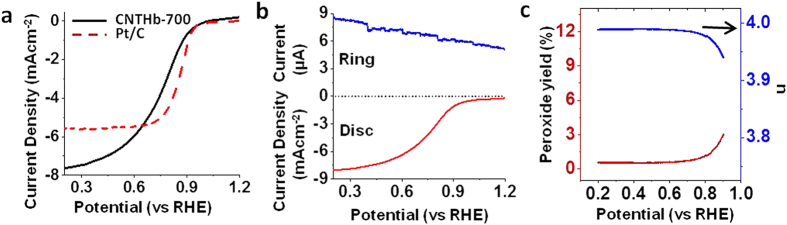
ORR polarization and RRDE. (**a**) Comparison of ORR polarization curves of **CNTHb-700** catalyst (300 μg cm^−2^) and 20% Pt/C (250 μg cm^−2^) and (**b**) RRDE voltammograms in 0.1 M HClO_4_ at 1600 rpm. (**c**) Peroxide yield with respect to oxygen reduction product (Red) and number of electrons (n) involved in ORR at 1600 rpm.

The electrochemical activity of **CNTHb-700** as a cathodic material for ORR was studied by cyclic voltammetry (CV) and linear sweep voltammetry (LSV) using rotator disc electrode (RDE) in acidic and basic media. The CVs of **CNTHb-700** in nitrogen saturated 0.1 M HClO_4_ and 0.1 M KOH solutions do not show any significant oxygen reduction peak which can be easily observed when same solutions were saturated with oxygen by purging it for 30 mins (Supporting Information Fig. S8a,b). It exhibits the ORR catalytic activity of **CNTHb-700** as cathode in both acidic and basic media. However, oxygen reduction capabilities of this material were studied in detail by LSV measurements on RDE at 1600 rpm and compared with commercial 20% Pt/C at scan rate 10 mV s^−1^ in oxygen saturated 0.1 M HClO_4_ and 0.1 M KOH solutions. In acidic medium, the polarization curve of **CNTHb-700** at 1600 rpm shows the onset potential of 0.92 V which is comparable to that of commercially available Pt/C under same conditions (Fig. 3a). Its half wave potential is just 75 mV lower than that of Pt/C. In basic medium under similar conditions, the onset potential of 0.92 V was observed (Supporting Information Fig. S9a). However, its half wave potential is ~55 mV lower than that of Pt/C. The material shows very high limiting current density of 6.34 and 6.69 mA cm^−2^ at 0.55 V (Fig. 3a, Supporting Information Fig. S9a) for acidic and basic media, respectively.

This catalytic performance of **CNTHb-700** can be attributed to (a) uniformly adsorbed small Fe_5_C_2_ nanoparticles on the surface of CNTs which provide the metal based catalytic site for ORR, (b) polarizing nitrogens doped in carbon matrix of CNTs, which enhance the binding ability of neighboring carbon towards molecular oxygen to facilitate ORR and dramatically increase the conductivity of material by providing favorable channel for electron transfer and (c) high surface area of mesoporous M-N/C material which endows the system with highly available catalytic surface.

The oxygen reduction activity of **CNTHb-700**, in acidic medium, was compared with other CNT-Hb materials i.e. **CNTHb-I**, **CNTHb-II** and **CNTHb-III** which were prepared by same synthetic route but with different ratio of starting materials *i.e.* CNT:Hb (6:2, 6:3, 6:5, respectively). Interestingly, by changing the ratio of CHT:Hb from 6:4, the decrease in onset potentials as well as half wave potential was observed which certainly indicates that **CNTHb-700** shows best catalytic performance (Supporting Information Fig. S9b). The decrease in amount of Hb in o-CNT perceptibly decreases the metal based catalytic sites, resulting in low catalytic activity. On the other hand, increasing the amount of Hb presumably leads to enveloped binding sites of N-doped CNTs by a larger number of Fe_5_C_2_ particles to decrease oxygen binding and ORR activity. Relatively, no significant ORR properties were observed in the case of activated isolated precursors o-CNT-700 and Hb-700 and unactivated hybrid adducts o-CNTHb (Supporting Information Fig. S9b).

The rotator ring disc electrode (RRDE) experiment was performed to calculate number of electrons involved in oxygen reduction and percentage of H_2_O_2_ production during the process in acidic and basic media. The hydrogen peroxide yield and number of electrons involved in ORR were calculated from disk currents and ring currents (Fig. 3b and Supporting information Fig. S10) in 0.1 M HClO_4_ and 0.1 M KOH, repsectively. In acidic medium, the yield of H_2_O_2_ is less than 1% and average number of electrons is 3.99, whereas in basic medium, the yield of H_2_O_2_ is less than 2% and average number of electrons is 3.98 (Supporting Information Fig. S10). To further confirm the 4-electron process, the ORR polarization curves at different rotation speeds from 300 to 1600 rpm were recorded in 0.1 M HClO_4_ (Supporting Information Fig. S11a) and 0.1 M KOH (Supporting Information Fig. S12a). Using Koutecky-Levich equation and *J*^−1^ versus ω^−1/2^ plots at different voltages (0.75, 0.73, 0.70, 0.67, 0.65, 0.63 and 0.60 V) drawn from ORR polarization curves, the number of electrons were calculated (Supporting Information Figs S11b and S12b) which corroborates with results of RRDE calculations to confirm the 4 e^−^ mechanism of oxygen reduction in acidic as well as basic medium.

Generally, the activation at low temperature is helpful in better nitrogen doping of carbon matrix, whereas the high temperature activation results in better catalytic activation of material. Therefore, we optimized the pyrolysis temperature of o-CNTHb adduct by observing the effect of different activation temperatures (500, 600, and 800 °C) on ORR properties of material in 0.1 M HClO_4_. The **CNTHb-700** gives the best ORR properties among **CNTHb**-**500/600/700/800** (Supporting Information Fig. S13).

**Figure 4:**
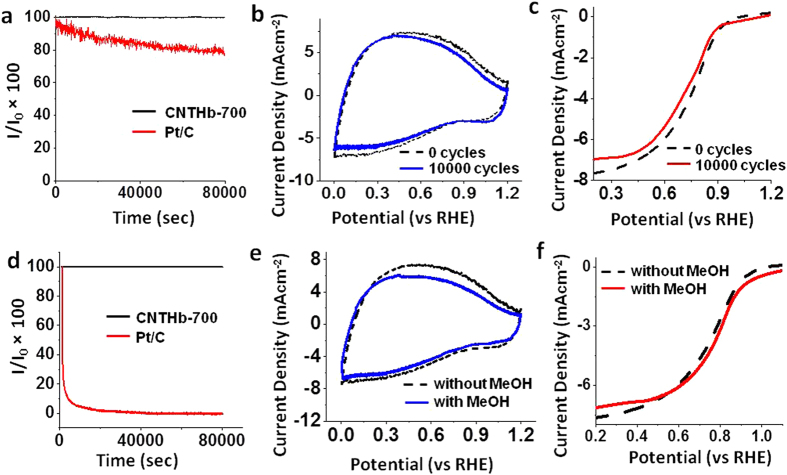
ORR catalytic Stability. (**a**) Chronoamperometric response obtained for **CNTHb-700** and 20% Pt/C at 0.55 V (*vs.* RHE) in 0.1M HClO_4_; I = final current intensity. (**b**) Cyclic voltammogram of **CNTHb-700** before and after 10000 cycles (scan rate 50 mVs^−1^) in 0.1M HClO_4_. (**c**) ORR polarization curves (scan rate 10 mVs^−1^) for **CNTHb-700** before and after 10000 cycles in 0.1M HClO_4_. (**d**) Chronoamperometric response for catalyst **CNTHb-700** and 20% Pt/C at −0.55 V in 1M MeOH + 0.1M HClO_4_; I = current density in 1M MeOH + 0.1M HClO_4_. (**e**) Cyclic voltammogram of **CNTHb-700** in the presence and absence of 1M methanol (scan rate 50 mVs^−1^) in 0.1M HClO_4_. (**f**) ORR polarization curves for **CNTHb-700** in the presence and absence of 1M methanol in 0.1M HClO_4_ (scan rate 10 mVs^−1^). RDE rotating speed was 1600 rpm; **CNTHb-700** catalyst loading was 300 μg cm^−2^. I_0_ = Initial current density without methanol.

The chronoamperometric study of **CNTHb-700** catalyst shows much higher stability in its current density than the Pt/C catalyst at 0.55 V (*vs.* RHE) in 0.1 M HClO_4_ (Fig. 4a). The accelerated degradation test (ADT) on cyclic voltammetry shows that **CNTHb-700** exhibits a high long term stability between 0–1.2 V in 0.1 M HClO_4_ (Fig. 4b). After 10000 cycles, no significant change in cyclic voltammogram of **CNTHb-700** was observed. In ORR polarization curve, after 10000 cycles, a small decrease of 30 mV in half wave potential was observed along with very small decrease in current density (Fig. 4c). However, no significant change in onset potential was observed. Likewise, **CNTHb-700** also exhibits very high stability in 0.1 M KOH (Supporting Information Figs S14 and S15). The referred very small decrease in limiting current and half wave potential is supposedly due to limited Ostwald ripening which results in redeposition of small Fe_5_C_2_ nanoparticles to form relatively bigger Fe_5_C_2_ nanoparticles, thus relatively reducing the number of metal based catalytic sites. This proposed hypothesis is supported by the particle size distribution calculation from HRTEM image of **CNTHb-700** which demonstrates that most of nanoparticles attain the diameter 10–15 nm after 10000 voltammetric cycles in 0.1M HClO_4_ (Supplementary Information, Fig. S16a,b). The XPS spectra of **CNTHb-700** after 10000 voltammetric cycles shows a relatively smaller but broader peak at 705.87 eV corresponding to the Fe-C bond, which confirms the stability of Fe_5_C_2_ nanoparticles (Supporting Information Fig. S16c).

Moreover, to study the practical utility of catalyst, the methanol tolerance of **CNTHb-700** was compared with that of Pt/C in acid and base. Upon adding 1 M of methanol in 0.1M of HClO_4_, no considerable change in current density was observed at 0.55 V (*vs.* RHE) for 80000 sec in the case of **CNTHb-700**, whereas a sudden drop in current density was observed in the case of Pt/C under similar conditions (Fig. 4d). Furthermore, no significant change in cyclic voltammogram and oxygen reduction polarization curve was observed in the case of **CNTHb-700** (Fig. 4e,f), whereas an intense inverse methanol oxidation peak was observed in ORR curve of Pt/C (Supporting Information Fig. S17) in acid. The catalyst **CNTHb-700** shows equally high methanol tolerance in 0.1 M KOH (Supporting Information Figs S18–S20), which is much superior to commercial Pt/C catalyst under similar conditions (Supporting Information Fig. S20a,b). These studies confirm much stable and selective catalytic behavior of **CNTHb-700** over commercial Pt/C.

Furthermore, to have a better insight of the role of heme groups in Hb in consequential high ORR activity by **CNTHb-700**, we synthesized two model cathodic materials **CNTCyt-700** and **CNTMy-700** by using other heme proteins with single heme group *i.e.* cytochrom-C and myoglobin, respectively, in place of hemoglobin. As per our anticipation, both materials show inferior ORR catalytic activity than **CNTHb-700** (Supporting Information Fig. S21) which is clearly due to the lesser available Fe_5_C_2_ based catalytic sites on nitrogen doped CNTs. FT-IR of **CNTMy-700** and **CNTCC-700** show much weaker stretching bands at 1197.98 and 1598.36 cm^−1^, and 1126.48 and 1553.62 cm^−1^, respectively, corresponding to C-N and C=N (Supporting Information Fig. S6). In addition to that, very weak diffraction peaks for Fe_5_C_2_ (510) can be observed in XRD spectra of **CNTMy-700** and **CNTCC-700**, which confirms the lesser available metal based catalytic sites (Supporting Information Fig. S5), and thus explains the low-grade ORR properties. This approach evidently confirms the role of heme groups, and the proposed mechanism of oxygen reduction by **CNTHb-700** as cathodic electrode. Thus, the Hb carrying four heme groups plays a pivotal role in providing uniformly distributed metal based catalytic sites on the surfaces of CNTs, whereas **CNTCC-700** and **CNTMy-700** carry single heme group each show inferior oxygen reduction properties, thus confirming the role of heme groups in synthesis of efficient catalyst.

## Discussion

The Fe_5_C_2_ nanoparticles have been generated by thermally activating commercially available lyophilized hemoglobin to prepare a metal coordinating nitrogen doped carbon supported highly mesoporous material **CNTHb-700**. This material behaves as an excellent cathodic catalyst for ORR with onset potential as good as the commercial catalyst Pt/C with much higher current density of 6.34 and 6.69 mA cm^−2^ at 0.55 V (vs. RHE) in 0.1 M HClO_4_ and 0.1 M KOH, respectively. The catalyst exhibits quite high stability as it retains its current density for 80000 sec at 0.55 V (vs. RHE) in chronoamperometric study in acidic as well as basic media. **CNTHb-700** exhibits much superior methanol tolerance than commercially available Pt/C which makes this catalyst a viable material for fuel cells.

## Methods

### Chemicals

Single walled carbon nanotubes were purchased from ILJIN Nanotech. Lyophilized hemoglobin (from human blood), myoglobin (from equine heart) and cytochrom C were purchased from Sigma Aldrich. The chemicals were used as such without any pretreatment. Double distilled water was used for synthesis and for electrochemical studies.

### General Synthesis of catalyst

The oxidized CNT (o-CNT) was synthesized by following the reported procedure^22^. To the aqueous dispersion of o-CNT (60 mg/50ml) was dropwise added the aqueous solution of desired ratio of hemoglobin Hb at 4 °C. The mixture was allowed to be stirred for 15 h at room temperature. The dispersed reaction mixture was centrifuged at 10000 rpm for 10 min. The resulting solid adduct o-CNTHb was washed several times with distilled water and dried. The o-CNTHb adduct was pyrolysed at desired temperature (9°/min) for 2h under NH_3_ gas. This activated material was leached by sonicating it in 0.1 M sulfuric acid for 3 h to give resulting catalyst.

### Characterization

Field-emission SEM (FE-SEM) measurements were carried out on Hitachi S-4800. HRTEM was performed on JEOL, JEM-2100F with an acceleration voltage of 200 kV. TEM samples were prepared by placing a drop of the colloidal solution on a carbon-coated copper grid and dried overnight at room temperature. Normal X-ray diffractometer (Normal-XRD) measurements were performed on a D8 advance (Bruker AXS). Raman spectroscopy was measured on WITec, Alpha-300R. Fourier transform infrared spectroscopy (FTIR) was measured on a VARIAN, 670/620. Inductively coupled plasma-optical emission spectrometer (ICP-OES) was carried out on VARIAN-700ES. Element analyzer (EA) measurements were performed on Flash 2000 (Thermo scientific, Netherlands). Magnetic measurements were performed on powder samples using a Quantum Design MPMS-XL SQUID magnetometer. XPS was measured on K-alpha (thermos fisher, UK). Brunauer-Emmett-Teller (BET) measurements were carried out on BELSORP-miniII (BEL Japan, inc).

### Electrochemical testing

All CVs were recorded by scanning the electrode potential from 0 to 1.2 V (*vs.* RHE) at a scan rate of 50 mV s^−1^. The VSP-Modular 2 Channels Potentiostat/Galvanostat/EIS (Bio-Logic Science Instruments) was calibrated with respect to an Ag/AgCl electrode. CV measurements were carried out in an N_2_-saturated 0.1 M HClO_4_ solution at room temperature. Rotating-disk electrode measurements were carried out in an O_2_-saturated 0.1M HClO_4_ solution at room temperature using a rotating-disk electrode system for ORR calibration. Linear sweep voltammograms were recorded by scanning the disk potential from 1.2 to 0.2 V (*vs.* RHE) at a scan rate of 10 mVs^−1^. For CV and rotating-disk electrode experiments, a three electrode configuration was used, consisting of a modified glassy carbon electrode (geometric area of 0.0707 cm^2^) as the working electrode, Ag/AgCl as reference electrode and Pt wire as a counter electrode. The voltammetric results were later converted with respect to standard hydrogen electrode. A 300 μg cm^−2^ of each synthesized catalysts and 250 μg cm^−2^ of Pt/C were loaded on glassy carbon electrode for all voltammetric analyses.

## Additional Information

**How to cite this article**: Vij, V. *et al.* Hemoglobin-carbon nanotube derived noble-metal-free Fe_5_C_2_-based catalyst for highly efficient oxygen reduction reaction. *Sci. Rep.* **6**, 20132; doi: 10.1038/srep20132 (2016).

## Supplementary Material

Supporting Information
