# Supplementary material for: Hemoglobin-carbon nanotube derived noble-metal-free Fe5C2-based catalyst for highly efficient oxygen reduction reaction
Source: Sci Rep. 2016 Feb 3;6:20132. doi: 10.1038/srep20132 (PMC4738279; doi:10.1038/srep20132)
Supplement: Supporting Information [file srep20132-s1.doc]

**Hemoglobin-carbon nanotube derived noble-metal-free Fe_5_C_2_-based catalyst for highly efficient oxygen reduction reaction**

Varun Vij, Jitendra N Tiwari,* Wang Geun Lee, Taeseung Yoon, Kwang S. Kim*

*Center for Superfunctional Materials, Department of Chemistry, Ulsan National Institute of Science and Technology (UNIST), UNIST-gil 50, Ulsan 689-798, Korea*

| **Page No.** | **Content** |
| --- | --- |
| **S3** | **Elemental Mapping of C , Fe and N, and Fe_5_C_2_ nanoparticles size distribution in CNTHb-700** |
| **S4** | **UV-vis spectra of o-CNT, Hb and o-CNTHb adduct, and Raman spectra of o-CNT, o-CNTHb, and CNTHb-700** |
| **S5** | **Powder X-ray diffraction pattern of CNTHb-700, CNTMy-700, CNTCC-700, and o-CNT and FT-IR spectra of o-CNT, CNTHb-700, CNTMy-700 and CNTCC-700** |
| **S6** | **Wide scan X-ray photoelectron survey spectrum and cyclic voltamogrammes of CNTHb-700 in N_2_ and O_2_ saturated solution of HClO_4_ and KOH** |
| **S7** | **ORR of CNTHb-700 and Pt/C in KOH, Comparison of ORR polarization curves of CNTHb-700 catalyst, 20% Pt/C, CNTHbI-III, o-CNTHb, o-CNT-700 and Hb-700, and RRDE experiment in 0.1 M KOH** |
| **S8** | **ORR polarization curves of CNTHb-700 catalyst at different RDE speeds and K-L plots drawn from ORR curves of CNTHb-700 at different potentials in 0.1 M HClO_4_ and KOH** |
| **S9** | **ORR polarization curves of CNTHb-700 catalyst as cathode at different activation temperatures in HClO_4_ and chronoamperometric response obtained for CNTHb-700 and 20 % Pt/C at 0.55 V** |
| **S10** | **cyclic voltammograms of CNTHb-700 before and after 10000 cycles in 0.1 M KOH and ORR polarization curves of CNTHb-700 before and after 10000 cycles in 0.1 M KOH; and HR-TEM image and XPS spectra of Fe(2p) of catalyst CNTHb-700 after 10000 cycles of cyclic voltammetry** |
| **S11** | **ORR polarization curves for 20% Pt/C in the presence and absence of MeOH in 0.1 M HClO_4_; and chronoamperometric response for catalyst CNTHb-700 and 20% Pt/C at -0.55 V in 1M MeOH + 0.1 M KOH** |
| **S12** | **CV of CNTHb-700 in the presence and absence of 1M methanol in 0.1 M KOH; and ORR polarization curves for CNTHb-700 in the presence and absence of 1M methanol in 0.1 M KOH and ORR polarization curves for 20% Pt/C in presence and absence of 1 M MeOH in 0.1 M KOH** |
| **S13** | **ORR polarization curves of CNTHb-700, CNTMy-700 and CNTCC-700 catalysts and calculation of no. of electrons involved in ORR.** |
| **S14** | **Preparation of working electrodes and RRDE parameters.** |

**a b**


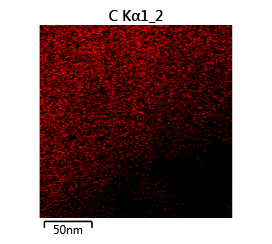

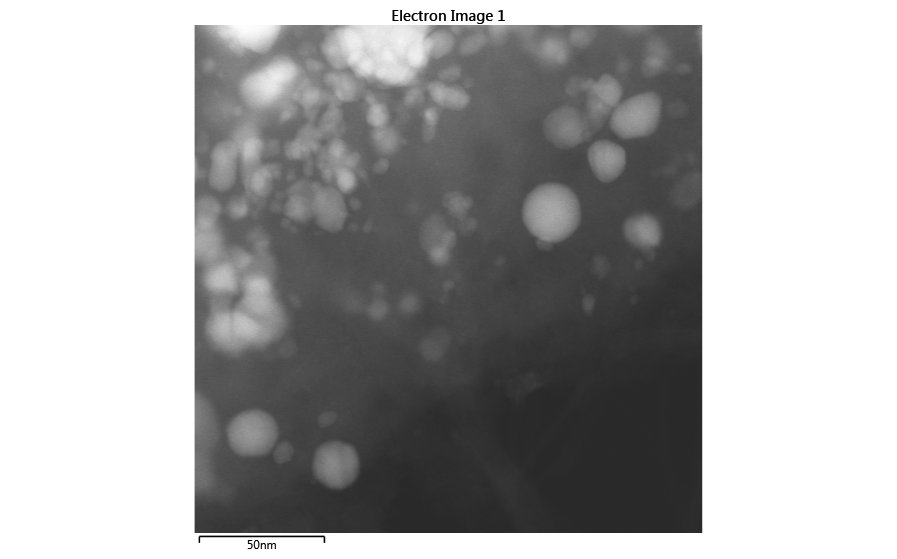


**50 nm**


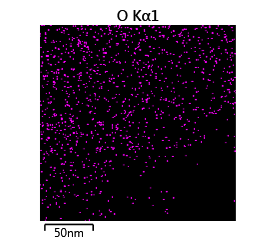


**c d e**


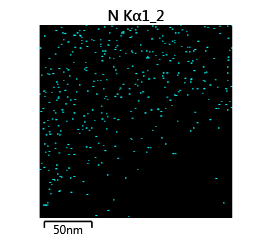

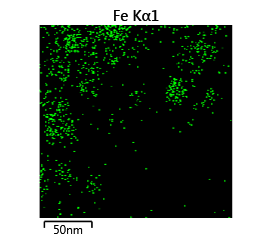


**Figure S1 | HRTEM image and elemental mapping. (a) HRTEM image of CNTHb-700 (b-e)** Elemental mapping of carbon (b), iron (b), nitrogen (c) and oxygen (e) of (a).

**0 5 10 15 20 25 30 40**

***d_p_* (nm)**

**% Frequency**

**b**

**a**


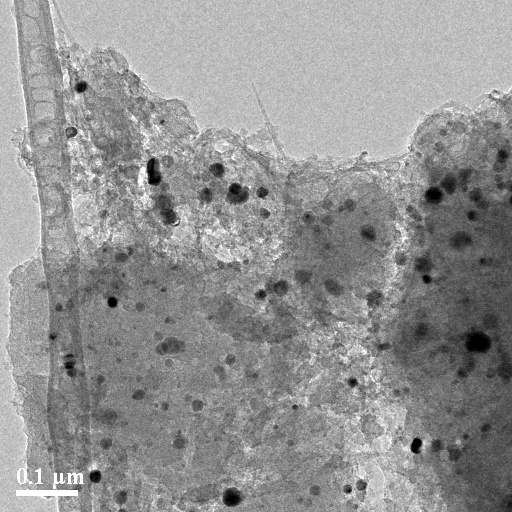


**50 nm**

**Figure S2 | Particles size distribution**. Fe_5_C_2_ nanoparticles size distribution in **CNTHb-700 (b)** calculated from TEM image (**a**).


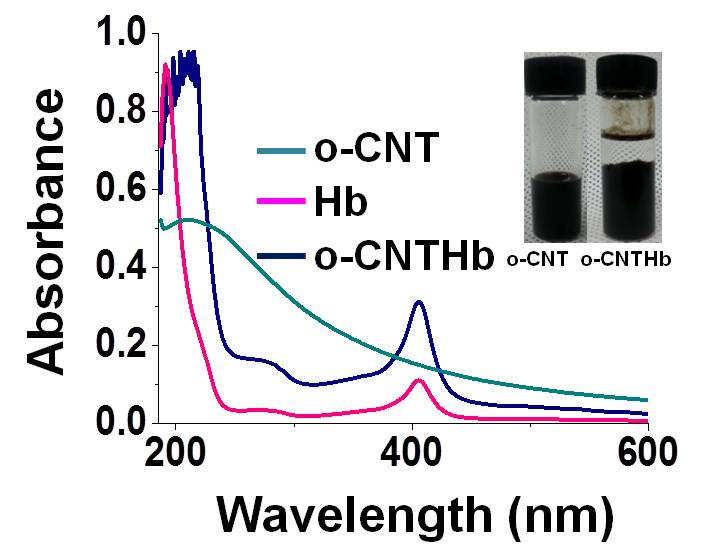


**Soret Band**

**Figure S3 | UV-Vis spectral study.** UV-vis spectra of o-CNT, Hb and o-CNTHb adduct. Inset shows the observed change in solution of o-CNT upon addition of Hb.


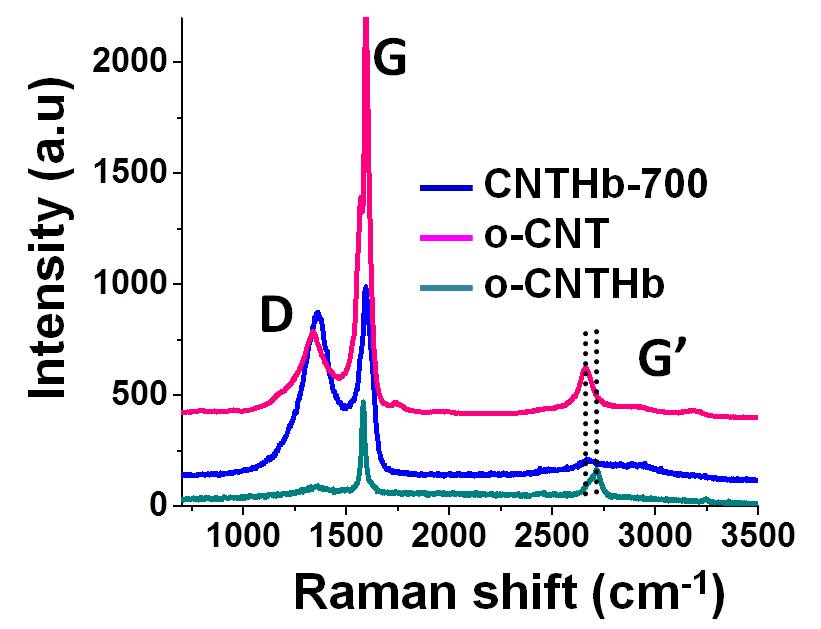


**Figure S4 | Raman spectra.** Raman spectra of **o-CNT, o-CNTHb,** and **CNTHb-700**


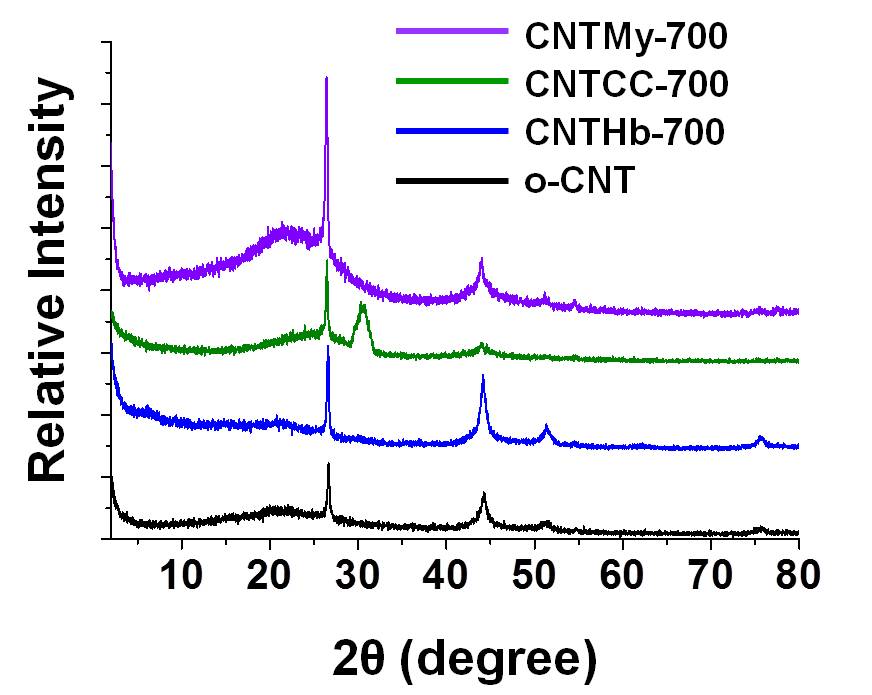


**Fe_5_C_2_ (510)**

**C(002)**

**Figure S5 |** **Characterization of diffraction pattern of materials.** X-ray diffraction pattern of **CNTHb-700, CNTMy-700, CNTCC-700,** and **o-CNT.**


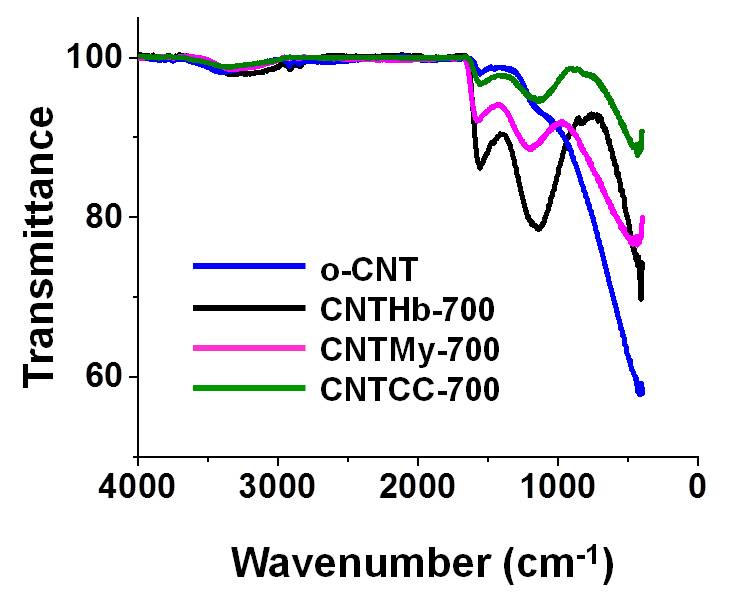


**Figure S6 |** **Characterization using** **FT-IR study.** FT-IR spectra of  **o-CNT, CNTHb-700, CNTMy-700** and  **CNTCC-700**.


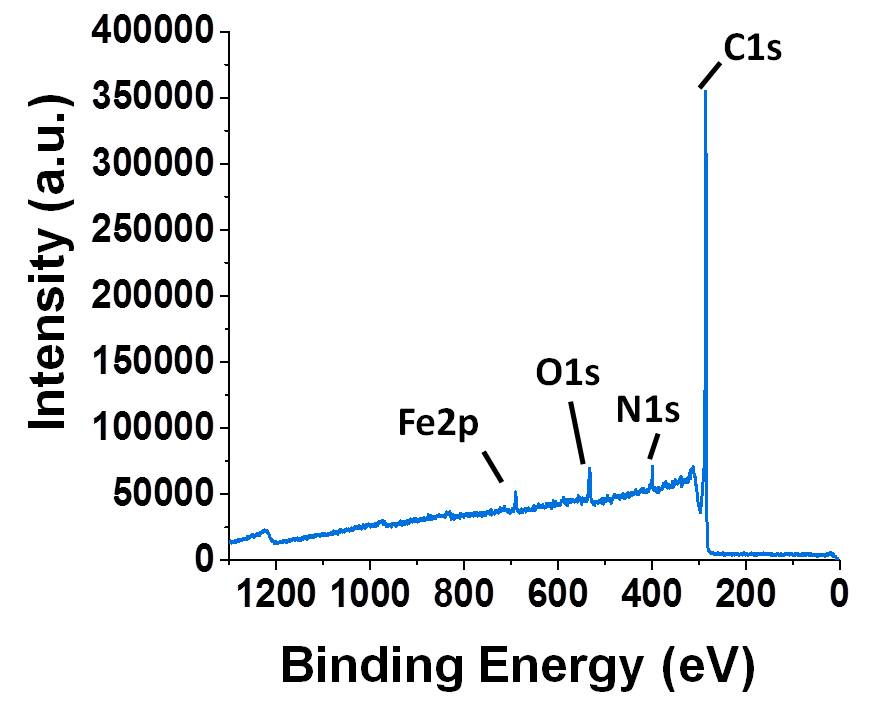


**Figure S7 | XPS spectra.** Wide scan X-ray photoelectron survey spectrum of **CNTHb-700** showing the peaks corresponding to C(1s), N (1s), O(1s) and Fe(2p).

**a**

**Potential (*vs.* RHE)**

**Current Density (mA cm^-2^)**

**N_2_ saturated**

**O_2_ saturated**

**b**

**Potential (*vs.* RHE)**

**Current Density (mA cm^-2^)**

**N_2_ saturated**

**O_2_ saturated**

**Figure S8 |** **ORR properties from cyclic voltammetry.** Cyclic Voltammograms of (**a**) **CNTHb-700** (as cathode) in N_2_ and O_2_ saturated 0.1 M HClO_4_. (**b**) **CNTHb-700** (as cathode) in N_2_ and O_2_ saturated 0.1 M KOH.

**a**

**CNTHb-700**

**Pt/C**

**Potential (*vs.* RHE)**

**Current Density (mA cm^-2^)**

**Current Density (mA cm^-2^)**

**Potential (*vs.* RHE)**

**CNTHb-III**

**CNTHb-II**

**CNTHb-I**

**CNTHb-700**

**20 wt% Pt/C**

**o-CNT-700**

**Hb-700**

**o-CNTHb**


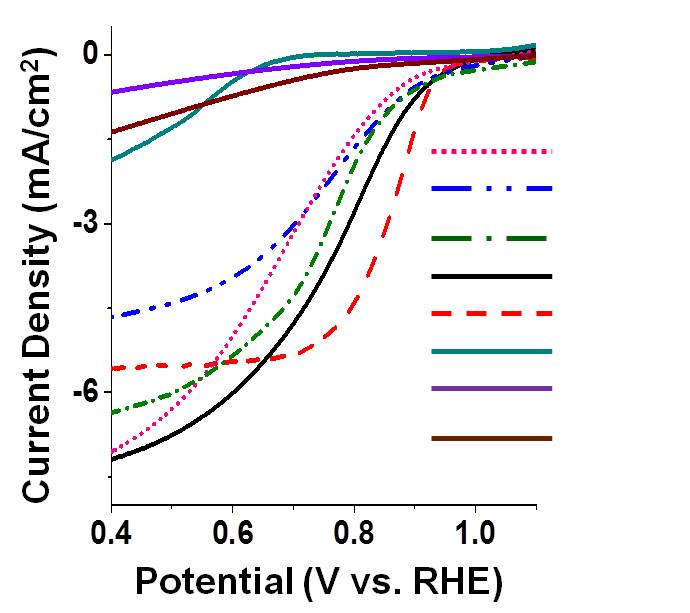


**b**

**Figure S9 |** **Comparison of** **ORR polarization curves.** ORR polarization curves of (**a**) **CNTHb-700** catalyst (300 µg cm^2-^) and 20% Pt/C in 0.1 M KOH. (**b**) **CNTHb-700** catalyst, 20% Pt/C, **CNTHbI-III** (6:2, 6:3 and 6:5), activated **o-CNT**, activated hemoglobin and inactivated **o-CNTHb** adduct at room temperature in 0.1 M HClO_4_; RDE rotating speed, 1600 rpm; catalysts loading 300 µg cm^-2^. Scan rate for all ORR analysis was 10 mVs^-1^.

**b**

**Potential (vs. RHE)**

**Peroxide Yield (%)**

**n**

**a**

**Ring**

**Disc**

**Potential (vs. RHE)**

**Current Density Current**

**(mAcm^-2^) (µA)**

**Figure S10 |** **RRDE analysis** (**a**) RRDE voltammograms in 0.1 M KOH at 1600 rpm. (**b**) Peroxide yield with respect to oxygen reduction product (Red) and number of electrons (n) involved in ORR at 1600 rpm.


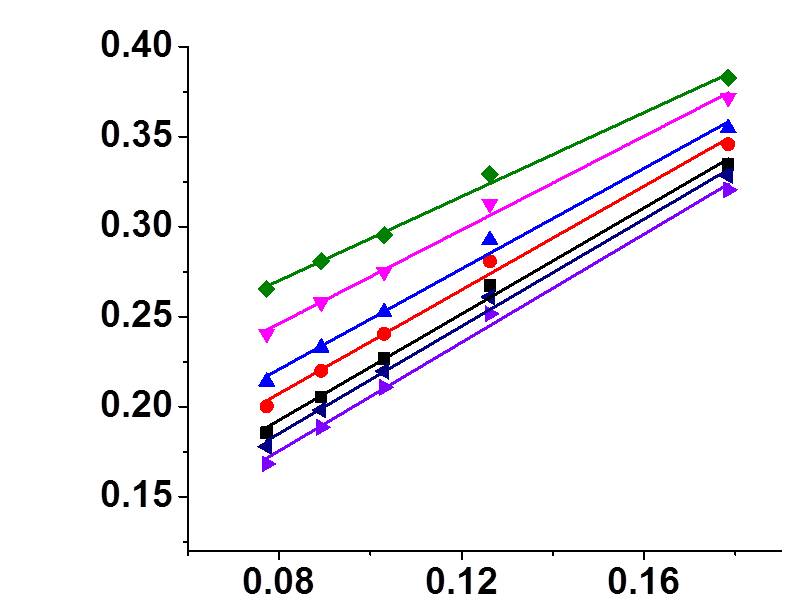


**-j^-1^ (mA^-1^cm^2^)**

**ω^-1/2^ (rad^-1/2^s^1/2^)**

**n= 3.9**

**b**


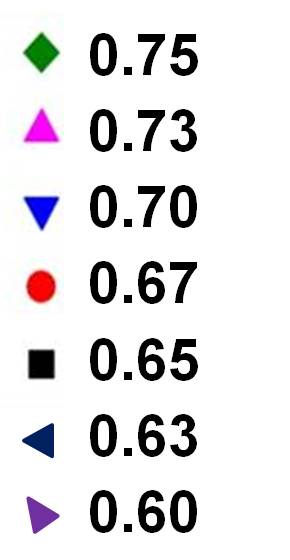


**a**

**Potential (*vs.* RHE)**

**Current Density (mAcm^-2^)**


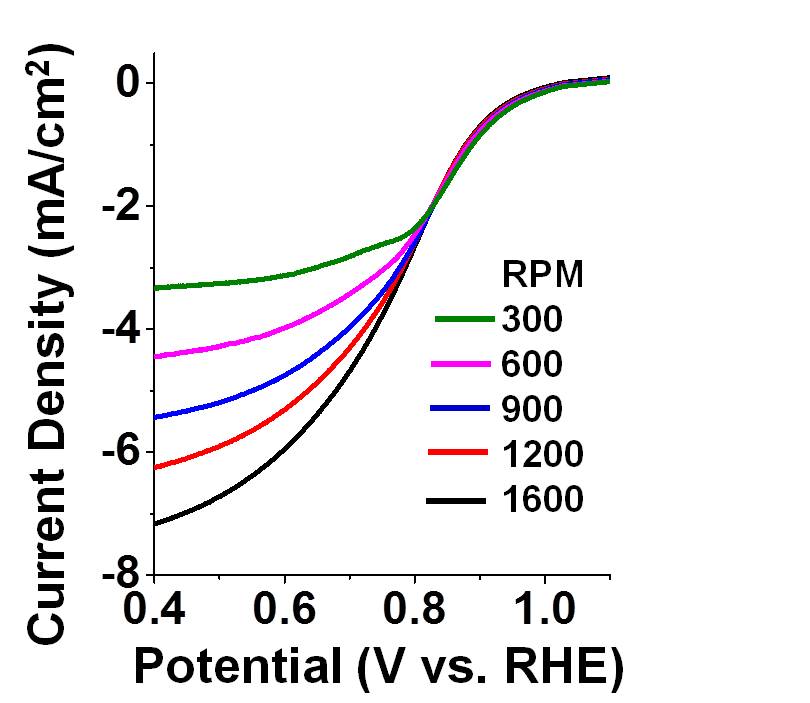


**Figure S11 |** **ORR catalytic mechanism.** (**a**) ORR polarization curves of **CNTHb-700** catalyst (300 µg cm^2-^) at different rotation speeds from 1600, 1200, 900, 600 and 300 rpm in 0.1 M HClO_4_. (**b**) Corresponding K-L plots drawn from ORR curves of **CNTHb-700** at different potentials in 0.1 M HClO_4_.

**a**


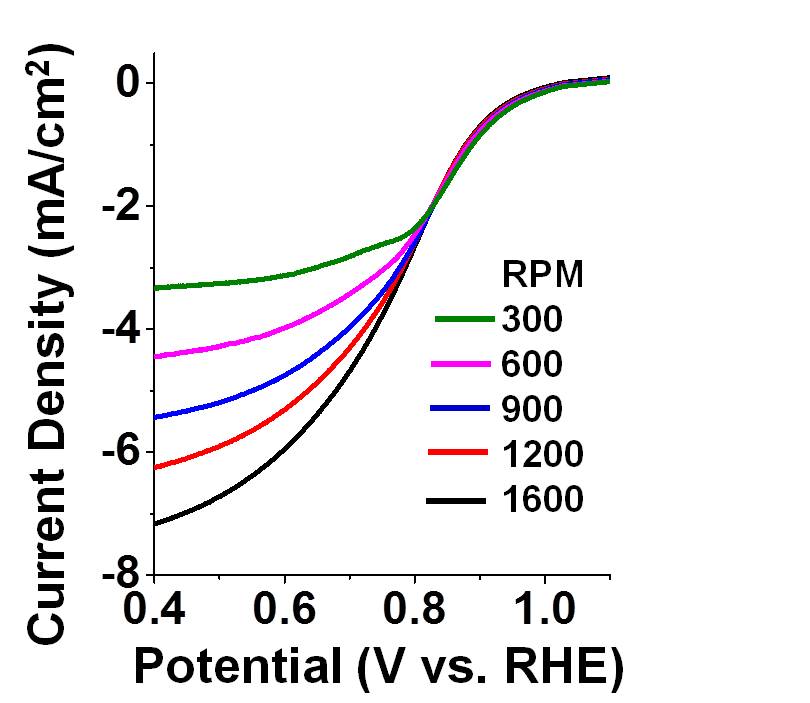


**Potential (*vs.* RHE)**

**Current Density (mA cm^-2^)**

**b**


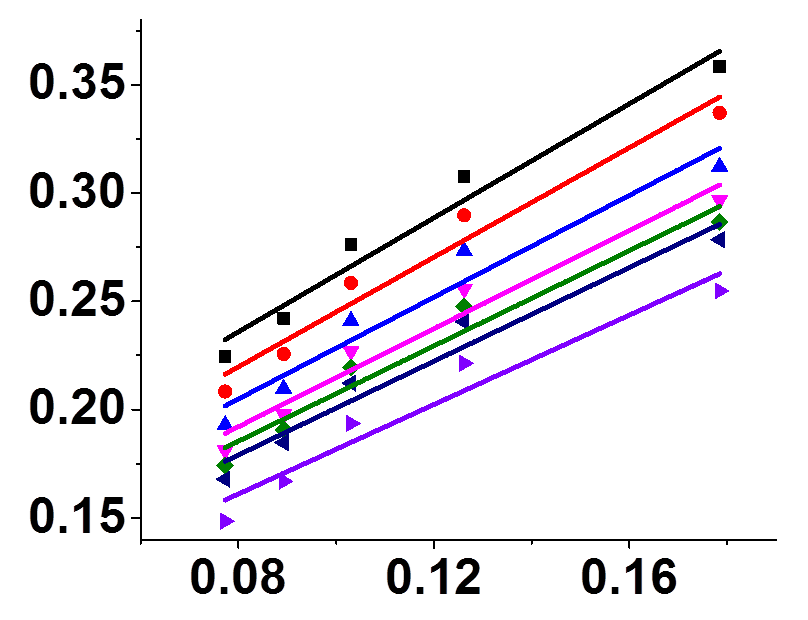


**-j^-1^ (mA^-1^cm^2^)**

**ω^-1/2^ (rad^-1/2^s^1/2^)**

**n= 3.9**


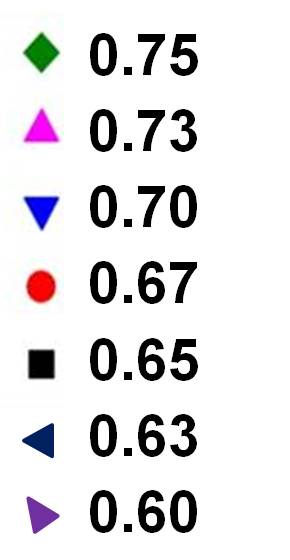


**Figure S12 |** **ORR catalytic mechanism.** (**a**) ORR polarization curves of **CNTHb-700** catalyst (300 µg cm^2-^) at different rotation speeds from 1600, 1200, 900, 600, and 300 rpm in 0.1 M KOH. (**b**) Corresponding K-L plots drawn from ORR curves of **CNTHb-700** at different potentials in 0.1 M KOH.


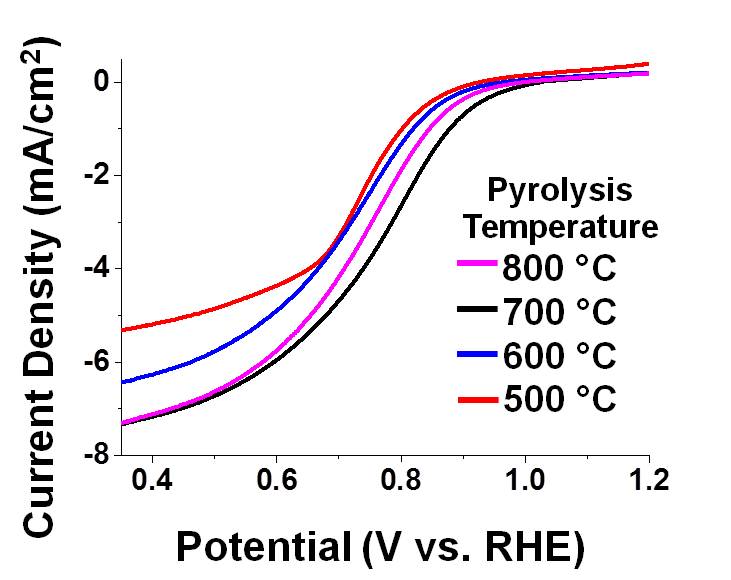


**Current Density (mA cm^-2^)**

**Potential (*vs.* RHE)**

**Figure S13 | Effect of pyrolyzing temperature on ORR.** ORR polarization curves of **CNTHb-700** catalyst as cathode at different activation temperatures. RDE speed 1600 rpm; catalyst loading 300 µg/cm^2^.

**Time (sec)**

**I/I_0_ × 100**

**CNTHb-700**

**Pt/C**

**Figure S14 | Stability of catalyst in basic medium.** Chronoamperometric response obtained for **CNTHb-700** and **20 %** **Pt/C** at 0.55 V (vs. RHE). RDE rotating speed was 1600 rpm; **CNTHb-700** catalyst loading was 300 µg cm^-2^, in the presence of O_2_ saturated 0.1 M KOH. I_0_ = Initial current density, I = Final current density.

**a**

**0 cycles**

**10000 cycles**

**Potential (*vs.* RHE)**

**Current Density (mA cm^-2^)**

**b**

**Potential (*vs.* RHE)**

**Current Density (mA cm^-2^)**

**0 cycles**

**10000 cycles**

**Figure S15 |** **Stability of ORR properties of catalyst**. (**a**) Cyclic voltammogram of **CNTHb-700** before and after 10000 cycles (scan rate 50 mVs^-1^) in the presence of O_2_ saturated 0.1 M KOH; RDE rotating speed was 1600 rpm; catalyst loading was 300 µg cm^-2^. (**b**) ORR polarization curves (scan rate 10 mVs^-1^) for **CNTHb-700** before and after 10000 cycles in 0.1 M KOH.

**0 5 10 15 20 25 30 40**

***d_p_* (nm)**

**% Frequency**

**b**


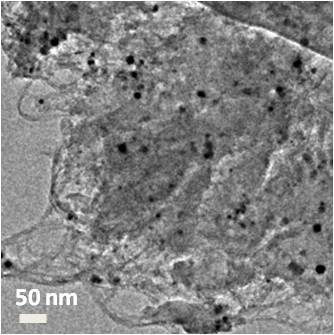


**a**

**50 nm**

**c**

**Binding Energy (eV)**

**Intensity (a.u.)**

**719.85 eV**

**Fe (2p)**

**711.10 eV**

**Fe (2p)**

**705.87 eV**

**Fe-C**

**Figure S16 | Effect of voltammetric cycles on morphology of catalyst. (a)** TEM image of catalyst **CNTHb-700** after 10000 cycles of cyclic voltammetry, (**b**) particles size distribution in TEM image, (c) XPS spectra of **CNTHb-700** after 10000 cyclic voltammetric cycles.

**without MeOH**

**with MeOH**

**Potential (*vs.* RHE)**

**Current Density (mA cm^-2^)**

**Figure S17 |** **Methanol tolerance of Pt/C.** ORR polarization curves for **20% Pt/C** in the presence and absence of 1M MeOH in 0.1 M HClO_4_. Scan rate was 10 mVs^-1^, RDE rotating speed was 1600 rpm.

**CNTHb-700**

**Pt/C**

**Time (sec)**

**I/I_0_ × 100**

**Figure S18 | Methanol tolerance.** Chronoamperometric response for catalyst **CNTHb-700** and **20% Pt/C** at -0.55 V in 1M MeOH + 0.1 M KOH. RDE rotating speed was 1600 rpm; **CNTHb-700** catalyst loading was 300 µg cm^-2^, in the presence of O_2_ saturated 0.1 M KOH. I_0_ = Initial current density without methanol, I = final current density in 1M methanol + 0.1 M KOH.

**without MeOH**

**with MeOH**

**Potential (*vs.* RHE)**

**Current Density (mA cm^-2^)**

**Figure S19 |** **Methanol tolerance** **in cyclic voltammetry**. Cyclic voltammogram of **CNTHb-700** in the presence and absence of 1M methanol (scan rate 50 mVs^-1^) in O_2_ saturated 0.1 M KOH. RDE rotating speed was 1600 rpm; Catalyst loading was 300 µg cm^-2^.

**b**

**Potential (*vs.* RHE)**

**Current Density (mA cm^-2^)**

**without MeOH**

**with MeOH**

**a**

**Potential (*vs.* RHE)**

**Current Density (mA cm^-2^)**

**without MeOH**

**with MeOH**

**Figure S20 |** **Methanol tolerance in ORR.** (**a**) ORR polarization curves for **CNTHb-700** in the presence and absence of 1M methanol (scan rate 10 mV s^-1^) in O_2_ saturated 0.1 M KOH. RDE rotating speed was 1600 rpm; and catalyst loading was 300 µg cm^-2^. (**b**) ORR polarization curves for **20% Pt/C** in the presence and absence of 1 M MeOH in 0.1 M KOH. Scan rate was 10 mV s^-1^, catalyst loading was 300 µg cm^-2^, and RDE rotating speed was 1600 rpm.


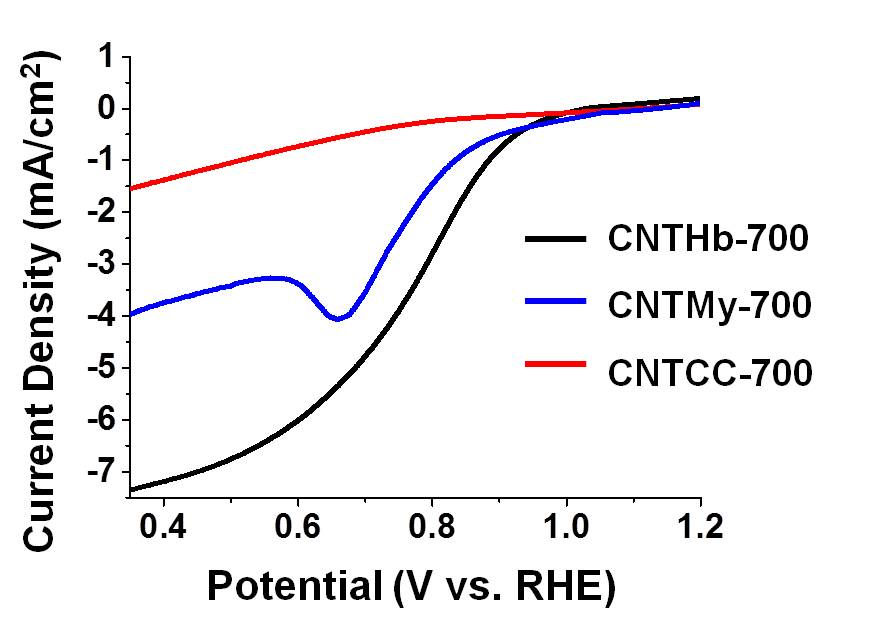


**Current Density (mA cm^-2^)**

**Potential (*vs.* RHE)**

**Figure S21 |** **Effect of number of Hb groups in ORR properties.** ORR polarization curves of **CNTHb-700**, **CNTMy-700** and **CNTCC-700** catalysts in 0.1 M HClO_4_; Scan rate was 10 mV s^-1^, catalyst loading was 300 µg cm^-2^, and RDE rotating speed was 1600 rpm.

**Calculation of No. of electrons involved in ORR.**

Koutecky–Levich (K-L) curves (J^-1^ vs. w^-1/2^) for the catalyst samples were analyzed at different potentials (Fig. 3c). The slopes of their best linear fit lines were used to evaluate the number of electrons transferred (n) on the basis of the K-L equation:

1/j =1/j_k_ + 1/j_d_ = 1/j_k_ + 1/Bw^1/2^

in which B=0.62nFAC_O2_D_O2_^2/3^/η^1/6^

where j is the experimentally obtained current, j_k_ is the kinetic current, j_d_ is the diffusion- limiting current, n is the number of electrons transferred, F is Faraday’s constant (F = 96485.34 C/mol), A is the electrode’s geometric area (A = 0.0707 cm^2^ ), C_O2_ is the O_2_ concentration in the electrolyte (Co_2_=1.26 x 10^-3^ mol/L), D_O2_ is the diffusion coefficient of O_2_ in the HClO_4_ solution (D_O2_ = 1.93 × 10^-5^ cm^2^/s), and η is the viscosity of the electrolyte (η = 1.009 × 10^-2^ cm^2^/s). The kinetic current was calculated based on the following equation:

J_k_ = (j x j_d_)/(j_d_-j).

**Preparation of working electrodes.** 7 mg of catalyst was dispersed in a 1 ml DI water using sonication. 3 μl of the dispersed sample solutions were then transferred onto the glassy carbon rotating-disk electrode with a geometric area of 0.0707 cm^2^ . The 4 μl of 0.05 wt% Nafion solution was added on top to fix the catalyst. Loading amount of catalyst is calculated by following method: Concentration of catalysts x loading of catalysts on RDE x wt% of catalysts For synthesized catalysts: 7 mg x 3 μl / 1000 µl / 0.0707 cm^2^  ~ 300 µg cm^-2^.

For Pt/C: 7 mg x 2.5 μl / 1000 µl / 0.0707 cm^2^  ~ 250 µg cm^-2^

**Rotating ring-disk electrode (RRDE) experiment**. Before the experiments, the Pt ring electrode was activated through CV in 0.5 M HClO_4_ from 0 to 1.4 V vs. RHE at a scan rate of 100 mV s^−1^ for 15 minutes. The disk electrode carrying **CNTHb-700** catalyst (300 µg cm^-2^) was scanned at a rate of 10 mV s^−1^, and the ring electrode potential was set to 1.2 V vs. RHE for both 0.1 M KOH and 0.1 M HClO_4_ at 1600 rpm. The hydrogen peroxide yield (% H_2_O_2_) and the electron transfer number (n) were calculated by the followed equations:

% H_2_O_2_=200 × (i_r_ / N) / [i_d_ + (i_r_ / N)]

N = 4 × i_d_ / [i_d_ + (i_r_ / N)]

Where i_d_ and i_r_ are the disk and ring currents, respectively. N is the ring current collection efficiency determined to be 37% by the reduction of 10 mM K_3_[Fe(CN)_6_] in 0.1 M KNO_3_.
